# Presenilin/γ-Secretase Regulates Neurexin Processing at Synapses

**DOI:** 10.1371/journal.pone.0019430

**Published:** 2011-04-29

**Authors:** Carlos A. Saura, Emilia Servián-Morilla, Francisco G. Scholl

**Affiliations:** 1 Institut de Neurociències, Departament de Bioquímica i Biologia Molecular, Centro de Investigación Biomédica en Red Enfermedades Neurodegenerativas (CIBERNED), Universitat Autònoma de Barcelona, Bellaterra, Barcelona, Spain; 2 Departamento de Fisiología Médica y Biofísica, Universidad de Sevilla, Instituto de Biomedicina de Sevilla, Sevilla, Spain; Institut National de la Santé et de la Recherche Médicale, France

## Abstract

Neurexins are a large family of neuronal plasma membrane proteins, which function as trans-synaptic receptors during synaptic differentiation. The binding of presynaptic neurexins to postsynaptic partners, such as neuroligins, has been proposed to participate in a signaling pathway that regulates synapse formation/stabilization. The identification of mutations in neurexin genes associated with autism and mental retardation suggests that dysfunction of neurexins may underlie synaptic defects associated with brain disorders. However, the mechanisms that regulate neurexin function at synapses are still unclear. Here, we show that neurexins are proteolytically processed by presenilins (PS), the catalytic components of the γ-secretase complex that mediates the intramembraneous cleavage of several type I membrane proteins. Inhibition of PS/γ-secretase by using pharmacological and genetic approaches induces a drastic accumulation of neurexin C-terminal fragments (CTFs) in cultured rat hippocampal neurons and mouse brain. Neurexin-CTFs accumulate mainly at the presynaptic terminals of PS conditional double knockout (PS cDKO) mice lacking both *PS* genes in glutamatergic neurons of the forebrain. The fact that loss of PS function enhances neurexin accumulation at glutamatergic terminals mediated by neuroligin-1 suggests that PS regulate the processing of neurexins at glutamatergic synapses. Interestingly, presenilin 1 (PS1) is recruited to glutamatergic terminals mediated by neuroligin-1, thus concentrating PS1 at terminals containing β-neurexins. Furthermore, familial Alzheimer's disease (FAD)-linked PS1 mutations differentially affect β-neurexin-1 processing. Expression of PS1 M146L and PS1 H163R mutants in PS−/− cells rescues the processing of β-neurexin-1, whereas PS1 C410Y and PS1 ΔE9 fail to rescue the processing defect. These results suggest that PS regulate the synaptic function and processing of neurexins at glutamatergic synapses, and that impaired neurexin processing by PS may play a role in FAD.

## Introduction

Alzheimer's disease (AD) is an age-related neurological disorder characterized by progressive memory impairment and neurodegeneration. The majority of familial AD (FAD) cases are caused by autosomal dominant mutations in the presenilin genes. Presenilins (PS) are the catalytic components of γ-secretase, an aspartyl protease that cleaves a number of type I membrane proteins involved in essential cell functions [1], [2]. Cleavage of the β-amyloid precursor protein (APP) by PS/γ-secretase generates the β-amyloid (Aβ) peptides, the principal component of cerebral plaques that accumulate in AD brains [3]. FAD-linked PS mutations affect differentially the γ-secretase-mediated cleavages of APP or other substrates, altering the γ-cleavage that generates Aβ peptides of different lengths and reducing the ε-cleavage that generates the soluble C-terminal intracellular domains [4]. PS inactivation in glutamatergic neurons in *PS1*/*PS2* conditional double knockout (PS cDKO) mice results in synaptic plasticity and memory impairment [5], which indicates that PS are essential for neuronal function. The synaptic and memory deficits caused by loss of PS function are independent of Aβ accumulation and occur through a largely uncharacterized synaptic mechanism. It has been recently reported that PS regulate neurotransmitter release during synaptic transmission [6]. These observations have led to the hypothesis that loss of PS function may be a key process in the physiopathology of FAD [7].

Neurexins are type I neuronal proteins that regulate synapse assembly and maturation. Neurexins are encoded by three genes each giving rise to longer α-neurexins and shorter β-neurexins in hundreds of alternatively spliced isoforms at the extracellular domains [8]. Neurexins mediate synapse formation by trans-synaptic binding to several postsynaptic partners including neuroligins, LRRTMs and GluRδ2 [9]–[15]. In the neurexin-neuroligin complex, the binding of β-neurexins with neuroligin-1 (+B) isoforms mediates glutamatergic differentiation, whereas neuroligin-2 interacts with α- and β-neurexins and concentrates at GABAergic synapses [12], [16]–[19]. Despite the identification of a number of neurexin partners, the mechanisms by which neurexin function and recruitment at synapses is regulated are not completely known. Interestingly, mutations in the *NRXN1* gene have been associated with autism and other brain disorders [20]–[22], which indicates that neurexin dysfunction could underlie the molecular basis of some mental diseases [23].

We explored the possibility that neurexins could be a substrate for PS/γ-secretase. Here we describe that neurexins are sequentially processed by metalloprotease and PS/γ-secretase activities. Genetic inactivation of PS/γ-secretase leads to accumulation of neurexin C-terminal fragments (CTFs), the substrate of γ-secretase, mainly at presynaptic compartments and enhances neurexin accumulation at glutamatergic synapses mediated by neuroligin-1 (+B). Moreover, FAD-linked *PS1* mutants differentially affect the processing of β-neurexin-1. These results indicate an important role for PS in regulating the synaptic function of neurexins, and suggest that defects in neurexin signaling may be involved in synaptic deficits associated with loss of PS function.

## Materials and Methods

### Ethics Statement

Animal procedures were performed in accordance with institutional andnational guidelines following approval by the Animal Care and Ethical Committee of the Universitat Autònoma de Barcelona (protocol # CEEA601) and the Ethics Committee of the University of Seville (protocol #113).

### Antibodies and reagents

The following commercially available primary antibodies were used in this study: rat anti-HA (Roche); mouse anti-synaptobrevin2/VAMP2 (Synaptic Systems); rabbit anti-Presenilin 1 N-terminal (1–65), rabbit anti-mouse/rat Presenilin 1 (Ab-2; 303–316) and rabbit anti PS1 loop (263–407) (Merck) [24]; mouse anti-CASK, mouse anti-N-Cadherin (clone 32), mouse anti-Nicastrin (clone 35) and mouse anti-PSD95 (BD Biosciences); rabbit anti-α-actin and mouse anti-β-actin (AC-15) (Sigma-Aldrich) and mouse anti-synaptophysin (SVP-38; Sigma-Aldrich). Chicken anti-cyto-neurexin antibodies have been previously described [11]. Cross-absorbed secondary antibodies conjugated with Cy2, Cy3 and Cy5 were used in immunofluorescence experiments (Jackson ImmunoResearch). In experiments with chemical inhibitors cells were treated with 2.5 µM GM6001, 20 µM TAPI-1 and 5 µM GL189 (Calbiochem) and 1 µM N-[N-(3,5-difluorophenacetyl-L-alanyl]-S-phenylglycine t-butyl ester (DAPT; Sigma-Aldrich) for 14 hours.

### DNA and lentiviral constructs

For expression experiments in HEK293 cells, cDNAs were cloned in a pCAGGS vector under control of the chicken β-actin promoter. Transfected rat α- and β-neurexin isoforms contained a HA-tag after the signal sequence. The neuroligin-1 splice isoform used in this study contained A and B insertions and a HA-tag after the signal sequence (named in the text as to HA-Nlg1), as previously described [25]. In β-neurexin-1 ΔC mutant, amino acids 413–468 corresponding to the cytoplasmic tail were deleted. Expression plasmids encoding human PS1 and FAD-linked variants PS1 M146L, PS1 H163R, PS1 C410Y and PS1 ΔE9 were subcloned in *Kpn*I/*BamH*I sites of the pAG3Zeo vector under the control of the β-actin promoter [26]. PS1 D385A subcloned in *EcoR*I sites in pCDNA3.1 was kindly provided by Dr. P. Fraering (*École Polytechnique Fédérale* de Lausanne, Switzerland) [27]. For infection of cultured hippocampal neurons, cDNAs were inserted in a previously described lentiviral vector under the control of the synapsin promoter (Syn) and upstream of a WPRE element [28]. Lentiviral vectors used in this study were: Syn-GFP-WPRE; Syn-HA-βNrx1-WPRE; Syn-HA-βNrx1 ΔC-WPRE; Syn-PS1-WPRE and Syn-PS1 D385A-WPRE.

Lentiviral particles were generated as previously described [29]. Briefly, HEK293T cells were transfected using the calcium phosphate method with the lentiviral expression vector and two packaging vectors: pCAG-VSVg, a plasmid expressing the VSV-G envelope gene, and pCMVΔR8.91, a plasmid expressing the gag/pol genes. The supernatants containing the viral particles were collected 48–60 hours after transfection and concentrated by ultracentrifugation. For infection of hippocampal neurons, cultures were transduced at the indicated day in vitro (DIV) by adding concentrated lentivirus into the growing media.

### Cell culture and transfection

Hippocampal tissues dissected from embryonic day 18–19 rat brains were incubated with trypsin (Gibco) in Dulbecco's modified Eagle's medium with high glucose (DMEM; Invitrogen) for 15 minutes at 37°C and mechanically dissociated with a Pasteur pipette in DMEM medium supplemented with 10% fetal bovine serum (HyClone), 44.4 mM D−(+)−glucose (Sigma), 2 mM Glutamax (Gibco), and Penicillin/Streptomycin (100 units/ml and 100 µg/ml, respectively; Gibco). Dissociated cells were plated at a density of 32,000 cells/cm^2^ in 24-well plate. The medium was replaced 2 hours after plating with neurobasal medium (Gibco) supplemented with B27 (Invitrogen), 2 mM Glutamax and Penicillin/Streptomycin. Hippocampal neurons were transfected using Lipofectamine 2000 (Invitrogen) at 10–14 DIV and fixed 2 days after transfection.

HEK293 cells were obtained from the American Type Culture Collection and grown in DMEM supplemented with 10% fetal bovine serum, 2 mM Glutamax and Penicillin/Streptomycin. HEK293 cells were transfected using Lipofectamine (Invitrogen). The mixed culture assays were performed essentially as described previously [13]. Briefly, HEK293 cells were transfected with HA-Nlg1 or GFP. Twenty-four hours after transfection, the cells were trypsinized and resuspended in neurobasal medium supplemented with 2 mM Glutamax, and cells were added into 14 DIV hippocampal cultures at a density of 25,000 cells/cm^2^ and maintained in culture for 24 hours. Mouse embryonic fibroblasts (MEF) derived from PS1/PS2 (+/+; control) or PS−/− embryos have been previously described [30]. Cells were transiently transfected for 24–48 h with empty vector or pCAGGS-HA-βNrx1 and pAG3Zeo-PS1 variants (PS1, PS1 M146L, PS1 H163R, PS1 C410Y and PS1 ΔE9) by using Lipofectamine 2000.

### Mice and brain fractionation

Generation and characterization of PS1−/− and PS cDKO mice have been previously described [5], [31]. Control (*fPS1/fPS1; PS2*+/+) and PS cDKO (*fPS1/fPS1; PS2*−/−; *α-CaMKII-Cre)* mice were generated by crossing *PS1* cKO; PS2+/− (*fPS1/fPS1; PS2*+/−; α-*CaMKII-Cre*) with floxed *PS1*; PS2+/− (*fPS1/fPS1; PS2*+/−) mice. Age-matched littermate mice in a C57BL6/129 hybrid background were used for this study.

Synaptic fractionations from mouse forebrains were prepared at 4°C essentially as described [32], [33]. Adult mice (three individuals per group) were rapidly decapitated, and cortices were dissected and homogenized in ice-cold Buffer A (5 mM HEPES, pH 7.4, 0.32 M sucrose, 1 mM NaHCO_3_, 1 mM MgCl_2_, 0.5 mM CaCl_2_, 1 mM NaF and phosphatase and protease inhibitors (Roche) by using a Teflon-glass homogenizer (12 strokes). The homogenate corresponding to the total lysate was centrifuged (1,400 x *g* for 10 min) and the pellet (P1) was re-extracted with Buffer A, homogenized (three strokes) and centrifuged (710 x *g* for 10 min). The supernatants (S1 + S1′) were combined and centrifuged (13,800 x *g* for 10 min) to collect the pellet (P2). P2 was resuspended in 3 ml ice-cold Buffer B (6 mM Tris, pH 8.0, 0.32 M sucrose, 1 mM NaHCO_3,_ 1 mM NaF and phosphatase and protease inhibitors) with a Teflon homogenizer (six strokes) and loaded onto the top of a discontinuous sucrose gradient (0.85 M, 1 M and 1.2 M sucrose solution) and centrifuged at 82,500 x *g* for 2 h. The synaptosome fraction was collected from the interface of the 1 M–1.2 M sucrose layers, diluted in equal volume of Buffer C (12 mM Tris HCl, pH 8.0, 1% Triton X-100) and incubated on ice for 15 min. The suspension was spin at 32,800 x *g* for 1 h to obtain the supernatant (presynaptic fraction). The pellet containing the postsynaptic fraction was resuspended in cold-ice Buffer D (40 mM Tris, pH 8.0, 1% NP40). The protein concentration was determined with the BCA protein assay kit (Pierce).

### Biochemical analysis

HEK293 cells and hippocampal neurons growing on 24-well plates were lysed in lysis buffer (50 mM Tris HCl, pH 7.4, 1% Triton X-100, 100 mM NaCl, 5 mM MgCl_2_, 0.1% SDS) containing protease inhibitor mixture (Roche). Embryonic brain (E17.5) or cortices from 2 month-old mice were homogenized in RIPA-DOC buffer (50 mM Tris HCl, pH 7.4, 150 mM NaCl, 2.5 mM EDTA, 1% NP40, 0.5% sodium deoxycholate, 0.1% SDS, 1 mM, Na_3_VO_4_, 50 mM NaF, 1 mM phenylmethylsulfonyl fluoride) supplemented with protease and phosphatase inhibitors. When indicated, cell culture media were used as starting material in immunoprecipitation experiments performed with the HA antibody. The immune complexes were precipitated with the Protein G Sepharose (GE Healthcare) and washed with pre-cooled lysis buffer. Equivalent amounts of protein lysates were resolved on 8–14% SDS-PAGE gels and transferred to PDVF membranes (Millipore). Immunoblotting was performed with the following antibody concentrations: HA (1∶1,000), Presenilin 1 N-terminal (1∶5,000), Presenilin 1 Ab-2 (1∶500), CASK (1∶200), N-Cadherin (1∶1,000), PS1 loop (1∶5,000), Nicastrin (1∶1,000), synaptophysin (1∶120,000), PSD-95 (1∶2,000), cyto-neurexin (1∶250–1∶1,200), β-actin (1∶60,000) and α-actin (1∶2,000). Immunoreactivity was detected with secondary antibodies conjugated to horseradish peroxidase (1∶5,000; Jackson ImmunoResearch) and developed with ECL Plus (GE Healthcare) or ECL kit (Perkin Elmer) using a ChemiDoc XRS System (Bio-Rad). Protein images were captured and quantified with the ImageJ software within a linear range of detection for the ECL reagent.

### Immunofluorescence and image analysis

Cell cultures were fixed with a 4% paraformaldehyde in phosphate buffer containing 4% sucrose for 20 min. After fixation, the cultures were permeabilized and blocked with 5% fetal bovine serum in PBS containing 0.05% TritonX-100. Cells were incubated with primary and secondary antibodies for 1–2 hours in the same blocking solution. The following antibody concentrations were used: HA (1∶1,000), VAMP2 (1∶3,000), Presenilin 1 N-Terminal (1∶500), Presenilin 1 Ab-2 (1∶25) and Cyto-neurexin (1∶250). Cells were mounted using Fluoromount-G (SouthernBiotech).

The images were acquired on an Olympus Fluoview FV1000 confocal laser scanning microscope (objective: PLAPON 60X; numerical aperture 1,42) using the same settings for photomultiplier voltage, gain and offset of all images. Maximal projections of Z-stacked images were obtained and analyzed with ImageJ software. The recruitment of neurexins and PS1 was quantified as the relative number of pixels per cell area above a threshold value. Thresholds were set such that most of the specific signal was selected. Thresholding was applied to create a binary image containing the positive pixels per each marker. The GFP or HA-Nlg1 channels were used to create a mask covering the cell area by drawing an outline around the transfected cells. The number of positive pixels were normalized to the cell area and averaged in each experimental condition. Data were plotted as mean ± SEM. Statistical significance was determined by ANOVA with dependent *post hoc* Fisher's multiple comparison test.

## Results

### Shedding of neurexins by metalloprotease- and γ-secretase-dependent cleavages

**Figure 1:**
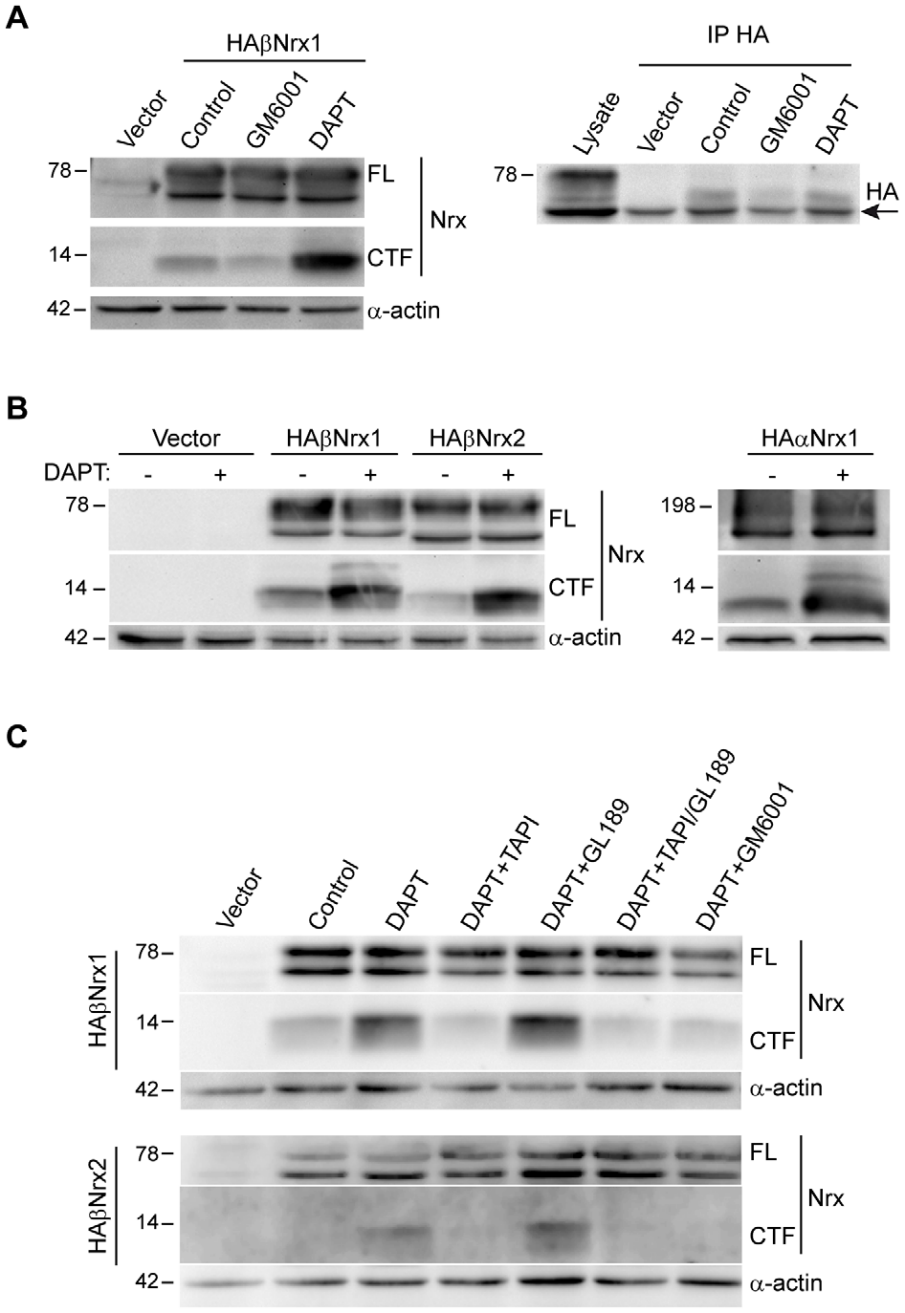
Processing of neurexins by metalloprotease- and γ-secretase-dependent cleavages. A) HEK293 cells were transfected with vector or HA-βNrx1 and treated with DMSO (control), GM6001 or DAPT, as indicated. Left panels: Western blot experiments of cell lysates analyzed with the cyto-neurexin antibody. Right panel: Western blot with an anti-HA antibody of HA-immunoprecipitates of conditioned media of transfected cells. A cell lysate of HEK293 cells transfected with HA-βNrx1 was included in the same blot to show βNrx-FL. Arrow points at IgG heavy chains in immunoprecipitates. B) HEK293 cells transfected with vector, HA-βNrx1, HA-βNrx2 or HA-αNrx1 were incubated with vehicle or DAPT, as indicated. The presence of neurexin-CTFs was analyzed by Western blotting with the cyto-neurexin antibody. C) COS cells transfected with HA-βNrx1 (upper panels) or HA-βNrx2 (lower panels) were incubated with DMSO (control) or with DAPT alone or in combination with TAPI-1, GL189, TAPI-1 and GL189, or GM6001 inhibitors, as indicated. Neurexin-CTFs levels were studied by Western blotting with the cyto-neurexin antibody. α-Actin levels were used as loading control.

The proteolytic processing of neurexin proteins was investigated in HEK293 cells, a mammalian cell line that expresses a functional active γ-secretase complex [34]. We transfected HEK293 cells with a β-neurexin-1 isoform containing a HA-tag at the N-terminal domain (HA-βNrx1). The expression of HA-βNrx1 was studied by Western blotting in cell lysates using two different antibodies: a HA antibody that recognizes the extracellular domain, and a cyto-neurexin antibody raised against the common cytoplasmic tail of neurexins [11]. The cyto-neurexin antibody detected two bands of ∼80 kDa and ∼50 kDa, corresponding to the glycosylated full-length β-neurexin-1 (βNrx-FL) and the unglycosylated core protein, respectively (Fig. 1A). Interestingly, the cyto-neurexin antibody detected two additional faint bands of ∼12–15 kDa in the same cell lysates, which were not observed with the HA antibody (Fig. 1A and data not shown). We speculated that the lower molecular weight bands could represent cleaved C-terminal fragments (neurexin-CTFs) generated by proteolytic shedding of the extracellular domain of HA-βNrx1. If so, such an activity might release the N-terminal extracellular domain (βNrx-NTF) to the media. Accordingly, immunoprecipitation experiments followed by Western blotting with the anti-HA antibody revealed the presence of βNrx-NTF in the media of HEK293 cells transfected with HA-βNrx1 (Fig. 1A). Interestingly, the apparent molecular weight of βNrx-NTF was ∼15 kDa lower than the βNrx-FL found in cell lysates (Fig. 1A), consistent with the generation of neurexin-CTFs by a proteolytic shedding of the mature protein.

Metalloproteases can proteolize the extracellular domain of a number of transmembrane proteins resulting in shedding of the soluble extracellular domain and the generation of membrane-bound CTF, which subsequently becomes a substrate for the PS/γ-secretase complex [35]. To analyze whether the processing of HA-βNrx1 required the activity of metalloproteases and PS/γ-secretase, we performed biochemical analysis in transfected cultures treated with GM6001, a broad metalloprotease inhibitor, or DAPT, a specific γ-secretase inhibitor. We found that GM6001 reduced both the levels of βNrx-NTF released to the media and neurexin-CTF found in cell lysates (Fig. 1A). Moreover, incubation with the PS/γ-secretase inhibitor DAPT increased basal accumulation of neurexin-CTFs in cell lysates, whereas it did not affect the generation of βNrx-NTF (Fig. 1A). To analyze whether other neurexin isoforms are processed by PS/γ-secretase, we transfected HEK293 cells with HA-αNrx1 or HA-βNrx2 and their processing was analyzed by Western blotting with the cyto-neurexin antibody. Similar to HA-βNrx1, expression of HA-αNrx1 or HA-βNrx2 induced the basal accumulation of neurexin-CTFs, which was further increased in cultures incubated with DAPT (Fig. 1B). Recently, it has been shown that the shedding of β-neurexin-3 ectodomain was inhibited by the TACE/ADAM17 inhibitor TAPI-1 [36]. We similarly found that TAPI-1, but not the BACE inhibitor GL189, inhibits the DAPT-induced accumulation of neurexin-CTF in HA-βNrx1- or HA-βNrx2-transfected cells (Fig. 1C). Together, these results suggest a sequential shedding of neurexin isoforms by metalloprotease- and γ-secretase-dependent activities and that inhibition of the PS/γ-secretase results in the accumulation of neurexin-CTFs.

### Accumulation of neurexin-CTFs in presenilin-deficient primary neurons

**Figure 2:**
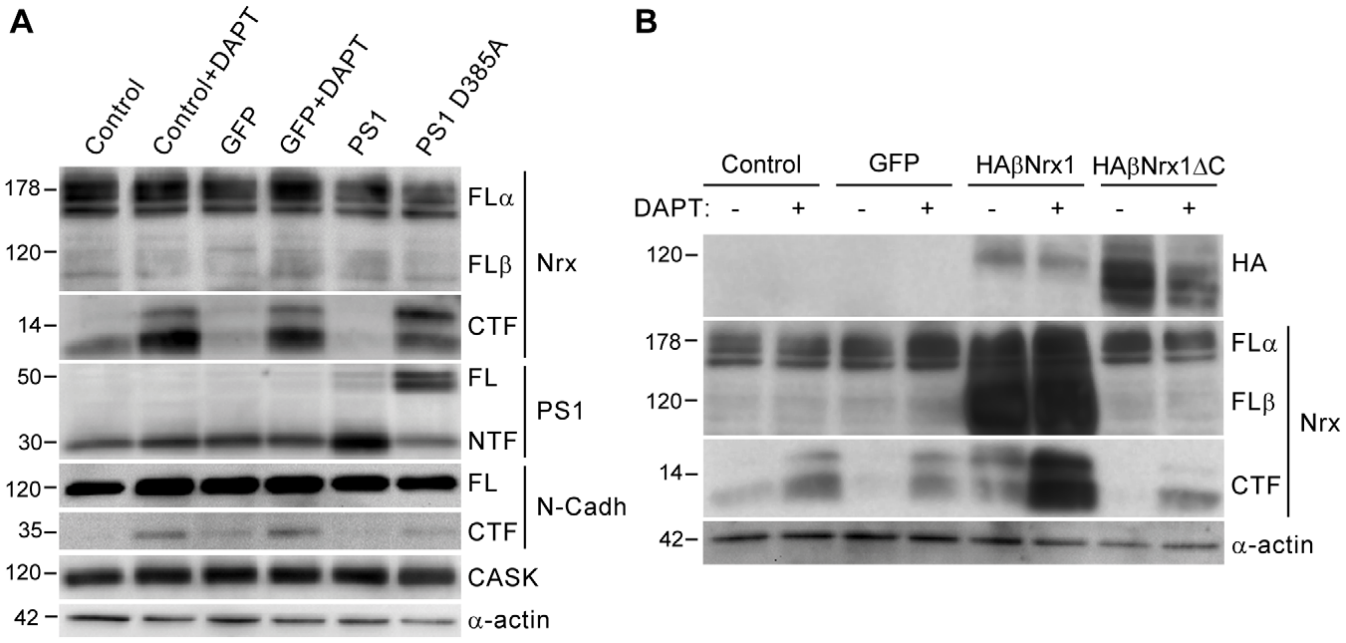
Accumulation of neurexin-CTFs in PS/β-secretase-deficient hippocampal neurons. A) Cultured hippocampal neurons (3–5 DIV) were non-infected (control) or infected with recombinant lentivirus (LV) expressing: GFP, PS1 or PS1 D385A. The levels of neurexins (Nrx), PS1, N-cadherin (N-cadh) and CASK were analyzed by Western blotting 9–10 days after infection. Non-infected cultures and cultures infected with GFP were treated with vehicle or DAPT for the last 14 hours. FLα: full-length α-neurexins; FLβ: full-length β-neurexins. B) Hippocampal neurons were non-infected or infected with LV-GFP, LV-HA-βNrx1 or LV-HA-βNrx1 ΔC and treated with vehicle or DAPT, as above. The expression of HA-tagged β-neurexin proteins and neurexin-CTFs was analyzed by Western blotting with an anti-HA and cyto-neurexin antibodies, respectively. α-Actin levels were used as loading control.

PS are the catalytic components of the γ-secretase, an enzymatic complex that cleaves the intramembrane domain of a number of signaling proteins [4]. The proteolytic activity of γ-secretase depends on two aspartate residues in PS1, D257 and D385, which form the catalytic site [27]. Thus, lack of PS1 or mutation of these residues result in the accumulation of γ-secretase substrates in neurons [27], [37], [38]. In order to study whether endogenous neurexins are processed by a PS/γ-secretase-dependent activity, we first analyzed the processing of neurexins in cultured hippocampal neurons treated with vehicle or DAPT. Biochemical analysis of hippocampal neurons with the cyto-neurexin antibody revealed a weak double band corresponding to endogenous neurexin-CTFs that were robustly increased by treatment with DAPT (Fig. 2A). Glycosylated full-length α- and β-neurexins were detected as bands of ∼150 and ∼100 kDa, respectively (Fig. 2A). As previously reported [39], N-cadherin-CTF was increased in DAPT-treated hippocampal neurons (Fig. 2A).

We next generated lentiviral vectors (LV) expressing human PS1 or PS1 D385A and performed a dominant-negative approach to block PS1 activity in hippocampal neurons. These lentiviral vectors contain the neuronal synapsin promoter, which directs the expression of the exogenous proteins to >90% of cultured neurons [28]. Infection of cultured hippocampal neurons with LV-PS1 D385A robustly and similarly increased the levels of neurexin-CTFs to the levels of control cultures treated with DAPT (Fig. 2A). In contrast, expression of LV-PS1 did not result in an increase of neurexin-CTFs. The expression of the exogenous PS1 was confirmed by Western blot experiments with an antibody that recognizes the N-terminal fragment of PS1. D385A mutation inhibits PS1 endoproteolysis resulting in an accumulation of the ∼50 kDa holoprotein [27]. As expected, expression of full-length PS1 concomitantly to a reduction in PS1-NTF was observed in PS1 D385A-infected neurons indicating an effective competition of the PS1 D385A mutant (Fig. 2A). As a control for the inhibitory effect on PS, we observed an accumulation of N-cadherin-CTF in cultures infected with LV-PS1 D385A (Fig. 2A).

In the above experiments, the proteolysis of specific neurexin isoforms could not be assessed since both α- and β-neurexins share common cytoplasmic tails, which are recognized by the cyto-neurexin antibody [11]. In an attempt to investigate whether β-neurexin proteins are processed by a PS/γ-secretase activity in neurons, we infected hippocampal neurons with recombinant LV expressing HA-βNrx1. As compared to non-infected cells or cells infected with GFP, overexpression of HA-βNrx1 increased the levels of neurexin-CTFs in both non-treated and DAPT-treated cultures (Fig. 2B). To confirm that the increase in neurexin-CTFs levels results from the processing of HA-βNrx1, rather than from the proteolysis of other neurexin isoforms, we expressed HA-βNrx1 ΔC, a mutant β-neurexin-1 protein that lacks the cytoplasmic tail and cannot be recognized by the cyto-neurexin antibody (Fig. 2B). Lentiviral overexpression of HA-βNrx1 ΔC did not result in an increase of neurexin-CTFs levels, as compared to HA-βNrx1 (Fig. 2B). Overexpression of HA-βNrx1 and HA-βNrx1 ΔC was confirmed by Western blot with the HA antibody (Fig. 2B). Altogether, these results indicate that β-neurexin isoforms are processed by a PS/γ-secretase–dependent cleavage, and that inhibition of PS/γ-secretase activity using pharmacological or genetic approaches results in the accumulation of neurexin-CTFs in neurons.

### Differential effect of FAD-linked *PS1* mutations in β-neurexin processing

**Figure 3:**
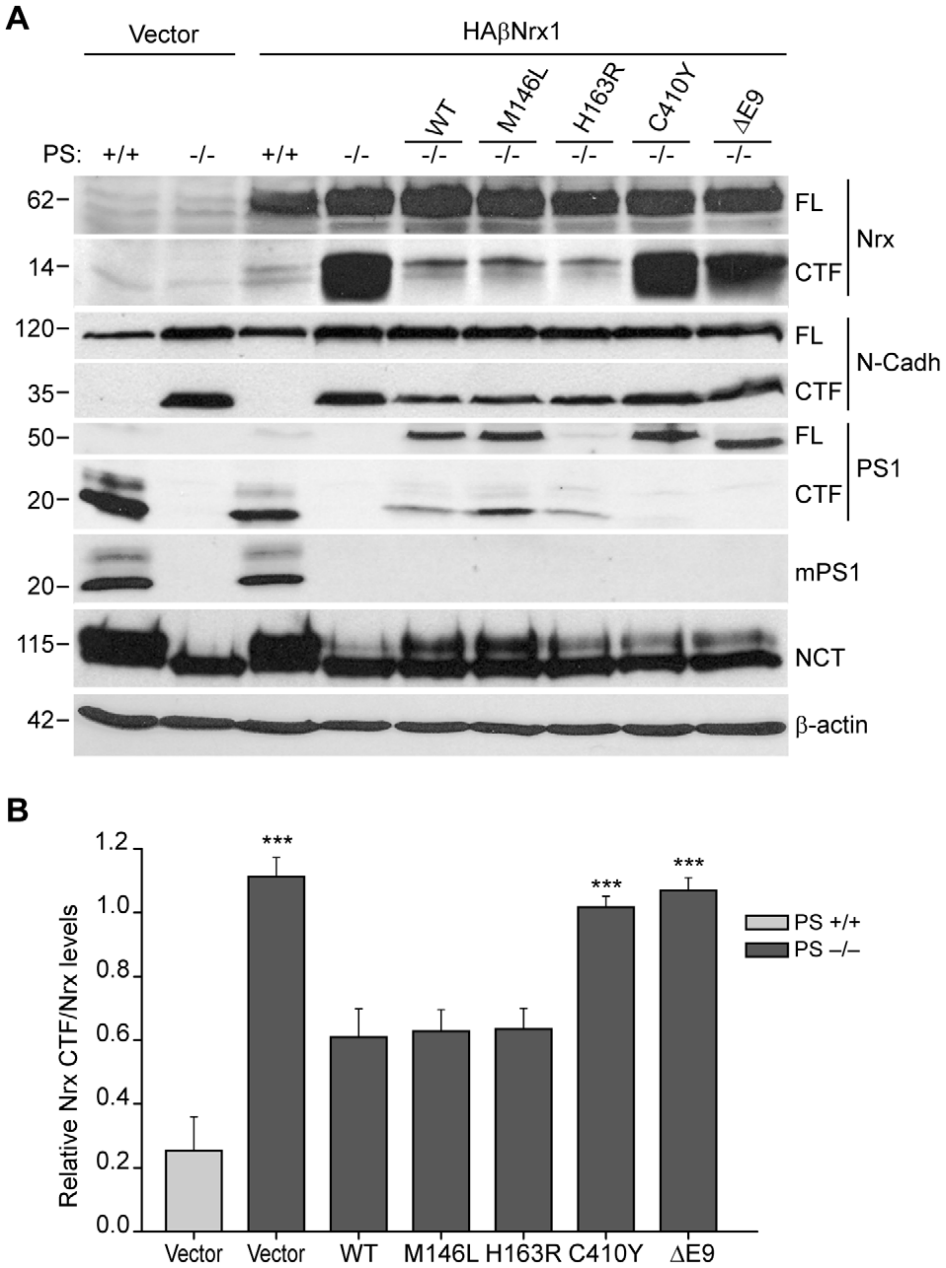
The processing of β-neurexin-1 is differentially affected by FAD-linked *PS1* mutants. A) PS+/+ or PS−/− MEF cells were transfected with vector or co-transfected with HA-βNrx1 and the following plasmids: empty vector, PS1 WT or the FAD-linked mutants PS1 M146L, PS1 H163R, PS1 C410Y or PS1 ΔE9. Cell lysates were subjected to Western blotting analysis with the cyto-neurexin (Nrx), N-cadherin (N-cadh), PS1 and Nicastrin (NCT) antibodies. To study PS1 expression, two different PS1 antibodies recognizing both mouse (m) and human PS1 (PS1 loop antibody) or mouse PS1 (Ab-2 antibody) were used. The endoproteolysis of PS1 (∼45 kDa) generates 18–20 kDa CTFs. The expression of PS1 WT and FAD-associated PS1 mutants increased the mature form of Nicastrin recognized as a slower migrating band in Western blot experiments. The FAD-linked *PS1* mutants C410Y and ΔE9 show deficient PS1 endoproteolysis and fail to rescue neurexin- and N-Cadherin-CTFs processing. β-Actin levels were used as loading control. B) Quantitative analysis of HA-βNrx1 CTFs relative levels. Data represents mean ± SD of three independent experiments. Statistical significant differences are shown (***P<0.001).

Autosomal dominant mutations in *PS1* and *PS2* genes cause the majority of FAD cases, although the mechanism by which these mutations cause memory deficits are largely unclear. To analyze the effect of FAD-linked *PS1* mutations in β-neurexin processing we used PS−/− MEF cells, which lack PS/γ-secretase activity [30]. HA-βNrx1 was transiently expressed in WT or PS−/− MEF cells together with wild-type PS1 or several FAD-linked PS1 variants. The ability of FAD-linked PS1 mutants to rescue the accumulation of neurexin-CTFs in PS−/− cells was analyzed by Western blotting. In agreement with the above data, expression of HA-βNrx1 led to a robust increase in neurexin-CTFs in PS−/− MEF cells (Fig. 3). Furthermore, co-expression of PS1 in PS−/− MEF cells efficiently reduced the accumulation of neurexin-CTFs to basal levels, confirming that PS1/γ-secretase activity is required for normal β-neurexin processing (Fig. 3). Interestingly, the PS1 M146L and PS1 H163R mutants efficiently rescued the normal processing of HA-βNrx1 to the levels of PS1 (Fig. 3). By contrast, expression of PS1 C410Y and PS1 ΔE9 mutants failed to rescue the normal processing of HA-βNrx1, despite they were expressed at similar levels than PS1 (Fig. 3). Similarly, expression of PS1 WT and the FAD-linked *PS1* mutants M146L and H163R decreased the accumulation of endogenous N-Cadherin-CTF in MEF PS−/− cells, while the expression of PS1 C410Y and PS1 ΔE9 did not alter N-Cadherin-CTF levels (Fig. 3). It is important to note that transient expression of PS1 WT and all FAD-linked *PS1* mutants increased to some extent the maturation of nicastrin, which indicates incorporation of the PS proteins into the γ-secretase complex. Notably, PS1 C410Y and PS1 ΔE9 FAD-linked mutants showed a significant reduction of autoproteolytic cleavage [40], as indicated by the presence of the unprocessed PS1 form of ∼50 kDa (Fig. 3), suggesting that the proteolytic processing of PS1 is required for the efficient cleavage of neurexin-CTFs. Together, these results indicate that some FAD-linked *PS1* mutations cause a loss of function effect in processing β-neurexins and suggest that the abnormal processing of β-neurexins might contribute to the synaptic defects associated with loss of PS function.

### Accumulation of neurexin-CTFs in synaptic fractions of PS cDKO mice

**Figure 4:**
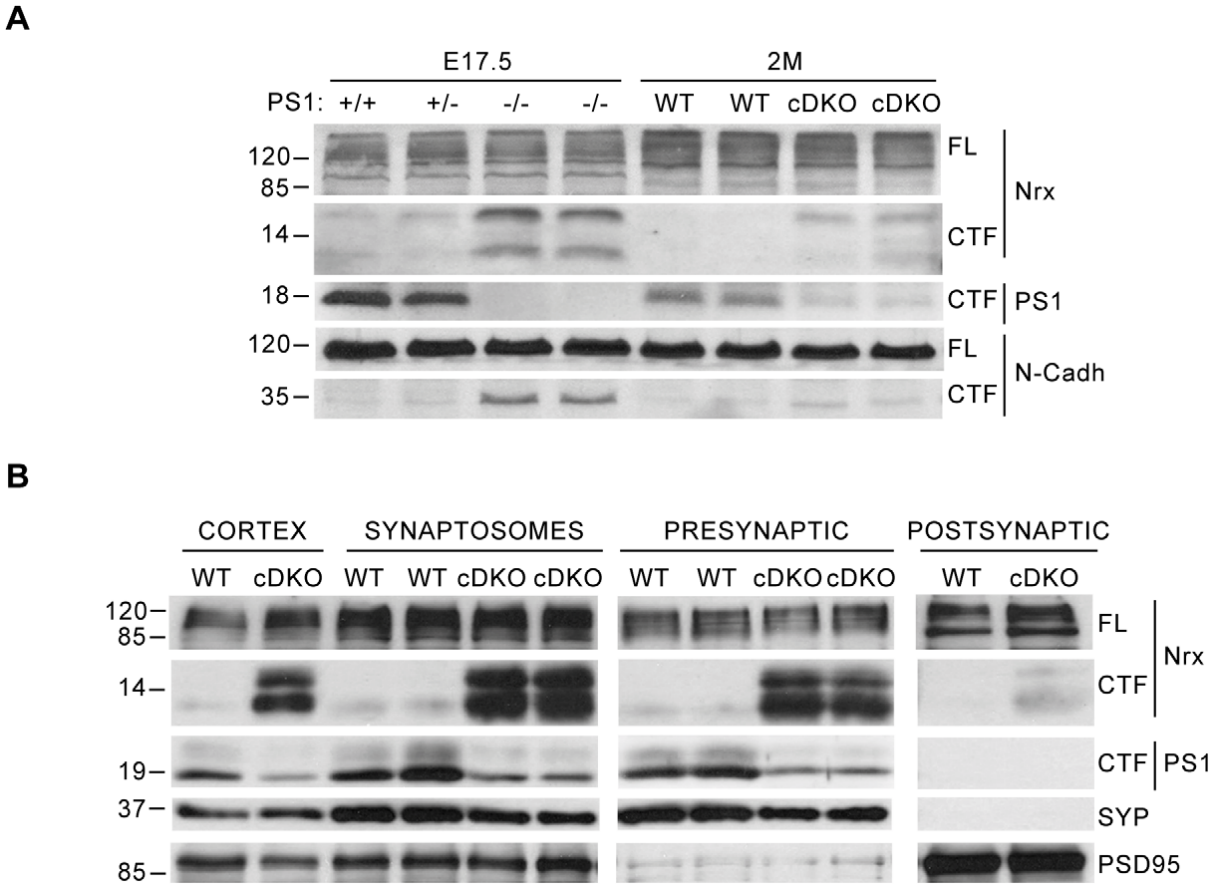
Accumulation of neurexin-CTFs in synaptic fractions of PS cDKO mice. A) Brain lysates from PS1+/+, PS1+/− and PS1−/− mouse embryos (E 17.5) or 2 month-old control wild-type (WT) and PS cDKO mice were analyzed by Western blotting with the cyto-neurexin (Nrx), PS1 and N-cadherin (N-cadh) antibodies, as indicated. Neurexin-CTFs and N-cadherin-CTF levels increased coinciding with decreased PS1 expression in animals lacking PS. The results were replicated in at least two independent mice per condition, as shown. B) Accumulation of neurexin-CTFs in purified synaptosomal and synaptic fractions from PS cDKO mice. Cortical brain lysates and pre- and post-synaptic fractions obtained from synaptosomal preparations from control wild-type (WT) and PS cDKO mice were analyzed by Western blotting with the cyto-neurexin, PS1, synaptophysin (SYP) and PSD95 antibodies. Neurexin-CTFs levels are markedly increased in synaptosomes and presynaptic fractions of PS cDKO mice. Synaptophysin and PSD-95 are specifically enriched in the presynaptic and postsynaptic fractions, respectively.

The above results point at a role of PS/γ-secretase-dependent processing of neurexins at synapses. To study the relevance of neurexin processing *in vivo* we examined endogenous neurexin processing in brains from PS1−/− embryos and adult PS cDKO mice. PS1−/− mice die shortly after birth, while PS expression in PS cDKO is selectively abolished in postnatal excitatory forebrain neurons allowing the study of PS in mature synapses [5], [31]. Consistent with a role of PS in the proteolysis of neurexins, we found a significant accumulation of neurexin-CTFs in brain lysates from PS1−/− embryos and PS cDKO adult mice compared with control littermate animals (Fig. 4A). Associated with decreased PS1 levels, N-cadherin CTF was also increased in PS1−/− and PS cDKO brains (Fig. 4A).

Previous results have demonstrated that PS localize at presynaptic terminals and that substrates of PS/γ-secretase complex, such as APP-CTF, accumulate in presynaptic terminals of PS knockout mice [6], [38]. To determine the subcellular localization of the accumulated neurexin-CTFs, we purified cortical synaptosomal fractions from control and PS cDKO brains and analyzed neurexin-CTFs and PS1 expression by Western blot analysis. Biochemical analyses revealed a specific accumulation of neurexin-CTFs coinciding with reduced PS1 levels in cortical synaptosomes of PS cDKO mice. The residual levels of PS1 in the cortex of PS cDKO mice are likely due to PS1 normal expression in glia and interneurons [5], [6]. Further subcellular fractionation showed that PS1 was localized mainly in the synaptophysin-enriched presynaptic fractions of control mice. Interestingly, neurexin-CTFs were mostly accumulated in the presynaptic fraction of PS cDKO mouse brains, although lower levels of neurexin-CTFs were also detected in the postsynaptic fraction (Fig. 4B). These results, which agree with our previous observations of accumulation of APP-CTF in synaptic terminals in PS1 cKO mice [38], indicate that PS regulates γ-secretase-dependent processing and accumulation of neurexin-CTFs at synaptic compartments.

### Expression of postsynaptic neuroligin-1 recruits PS1 to synaptic terminals

**Figure 5:**
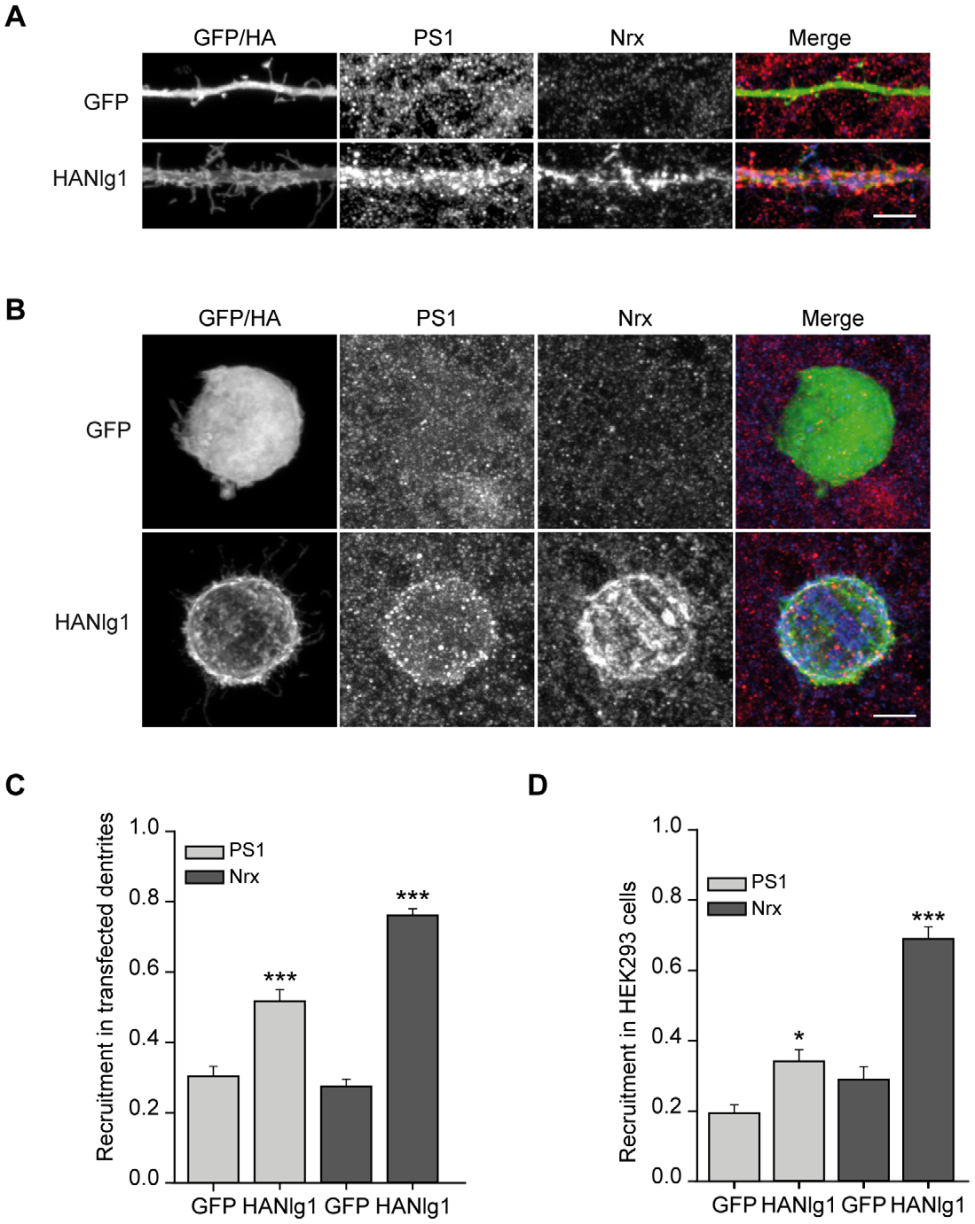
Recruitment of PS1 to neurexin/neuroligin mediated synapses. A) Confocal images of cultured hippocampal neurons transfected with GFP or HA-Nlg1 at 10–13 DIV and stained with PS1 (PS1 N-terminal antibody, red in the colocalization) and cyto-neurexin antibodies (blue in the colocalization) two days after transfection. PS1 is recruited along neurexins to HA-Nlg1 transfected dendrites. B) Hippocampal neurons (14–16 DIV) were co-cultured with HEK293 cells expressing GFP or HA-Nlg1 for 24 hours. The recruitment of PS1 and neurexins to the transfected HEK293 cells was analyzed by immunofluorescence with PS1 (Ab-2 antibody, red in the colocalization) and cyto-neurexin antibodies (blue in the colocalization). The Ab-2 antibody does not recognize human PS1 expressed in HEK293 cells. In A) and B) HA-Nlg1 expression was analyzed with a HA antibody and GFP expression was detected by direct fluorescence. C, D) Quantitative analysis of the recruitment of PS1 and neurexins to dendrites (C) or HEK293 cells (D) transfected with GFP or HA-Nlg1, as indicated. Recruitment was quantified as the relative area occupied by each specific marker in the transfected cell. Data collected from three (C, n = 25 cells per condition) or two (D, n = 20 cells per condition) independent experiments are shown. Asterisks indicate significant statistical differences (*P<0.05; ***P<0.001). Data are expressed as means ± SEMs. Scale bars: 5 µm.

Our data suggest that PS localizing at presynaptic terminals might mediate the proteolysis of neurexins at glutamatergic synapses. Therefore, we investigated whether PS are recruited to glutamatergic terminals mediated by the neurexin-neuroligin complex. With that purpose, we transfected cultured hippocampal neurons with HA-neuroligin-1 (+B) (named HA-Nlg1 for simplicity), a major neuroligin-1 isoform that triggers glutamatergic differentiation in hippocampal neurons [16]. Then, the recruitment of endogenous PS1 and neurexins to HA-Nlg1-induced synapses was analyzed by confocal microscopy. Consistent with previous results [11], we found that HA-Nlg1 triggered the recruitment of neurexins to the synaptic contacts (Fig. 5A). The recruited neurexins most likely correspond to β-neurexin isoforms, since HA-Nlg1 binds to β- but not α-neurexins [12], [14], [16]. When the distribution of PS1 was analyzed with an antibody recognizing the N-terminal domain, we found that PS1 was concentrated and partially overlapped with neurexins at HA-Nlg1-induced synapses (Fig. 5A). Moreover, PS1 partially colocalized with synaptic proteins, such as PSD95 and synaptobrevin, at HA-Nlg1 synapses (Fig. S1). Furthermore, quantification analysis of the relative dendritic area occupied by PS1 and neurexins revealed a significant increase in dendrites expressing HA-Nlg1 compared to dendrites expressing GFP (Fig. 5C). Moreover, PS1 intensity was also significantly increased on dendrites expressing HA-Nlg1 (114±5.3 in control GFP dendrites *vs* 130± 5.1 in HA-Nlg1 dendrites, p<0.05). Similar results were obtained with the Ab-2 antibody that recognizes the C-terminal domain of murine PS1 (data not shown). These results indicate that PS1 is accumulated at Nlg1-mediated synapses.

To analyze whether the recruitment of PS1 occurs at presynaptic terminals, we performed a mixed-culture assay of hippocampal neurons with HEK293 cells expressing HA-Nlg1. This assay allows the study of the recruitment of axonal markers in response to contact with non-neuronal cells expressing a specific neuroligin isoform [13], [41]. The localization of PS1 was analyzed with the Ab-2 antibody that only recognizes murine PS1 (see also Fig. 3). Therefore, recruitment of axonal PS1 to neuroligin-1 hemisynapses can be detected as enrichment of PS1 staining on HEK293 cells expressing HA-Nlg1. In HEK293 cells expressing GFP, the distribution of PS1 was similar in all areas of the field, regardless of the presence of HEK293 cells (Fig. 5B). In contrast, analysis of PS1 intensity (93±1.4 in control GFP cells *vs* 116±2.1 in HA-Nlg1 cells, p<0.001) and of the relative area occupied on the transfected cells indicated that PS1 was specifically recruited to HEK293 cells expressing HA-Nlg1 (Fig. 5B and 5D). Triple immunofluorescence staining and quantitative analyses revealed that axonal neurexins were also recruited to HA-Nlg1 hemisynapses (Fig. 5B and 5D). These data, which are consistent with the presynaptic localization of PS1 shown by biochemical experiments, strongly suggest that expression of HA-Nlg1 in dendrites triggers the recruitment of axonal PS1 at the presynaptic sites mediated by β-neurexins.

### PS1 regulates the recruitment of neurexins to neuroligin-1 synapses

**Figure 6:**
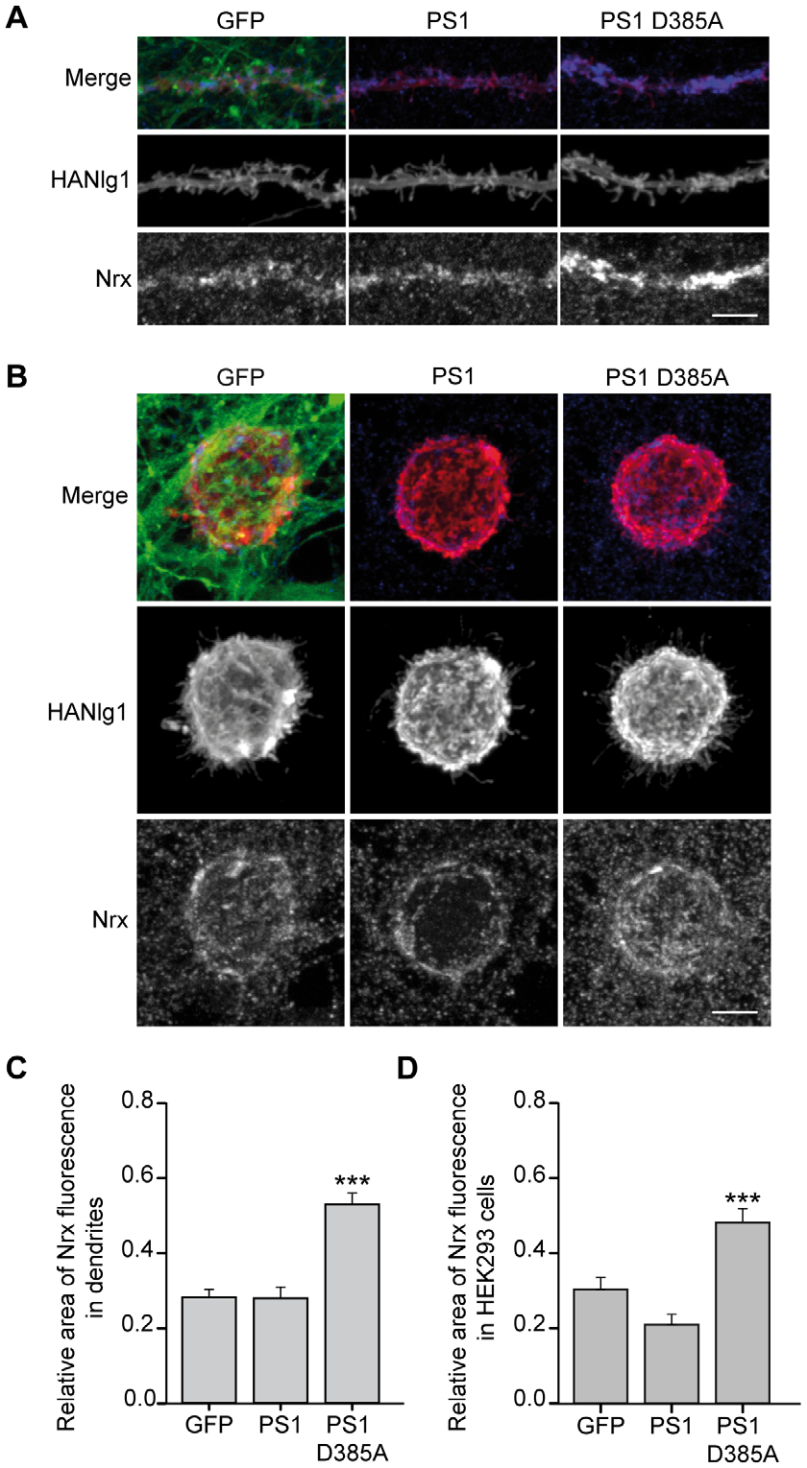
Recruitment of neurexins to HA-Nlg1-mediated synapses in PS1-deficient neurons. A) Confocal images showing the recruitment of neurexins to HA-Nlg1 synapses in hippocampal cultures overexpressing PS1 or PS1 D385. Hippocampal neurons were infected at 3 DIV with LV-GFP, LV-PS1 or LV-PS1 D385, as indicated. The cultures were transfected 9 days after infection (12 DIV) with HA-Nlg1 and the localization of HA-Nlg1 (middle panels, red in the colocalization), and neurexins (lower panels, blue in the colocalization) was analyzed by immunofluorescence 2–3 days after transfection. The fluorescence signal obtained with the cyto-neurexin antibody is increased in dendrites expressing PS1 D385. B) Recruitment of neurexins to HEK293 cells expressing HA-Nlg1 in neuronal cultures overexpressing PS1 proteins. Hippocampal neurons infected with LV-GFP, LV-PS1 or LV-PS1 D385 at 3 DIV were co-cultured at 12 DIV with HEK293 cells expressing HA-Nlg1 for 24 hours. The cultures were co-stained with HA and neurexin antibodies, as above. The expression of exogenous PS1 proteins in these hemisynapses is restricted to axons contacting HA-Nlg1 expressing cells. C, D) Quantification of neurexin recruitment to dendrites (C) or HEK293 cells (D) expressing HA-Nlg1. Pooled data collected from three independent experiments are shown (n>24 cells per condition). Data are expressed as means ± SEMs. ***P<0.001. Scale bars: 5 µm.

After showing that PS1 is concentrated at glutamatergic terminals mediated by HA-Nlg1, we investigated whether blocking PS function increases neurexin-CTFs levels at these terminals. To explore this possibility, we infected cultured hippocampal neurons with lentiviral vectors expressing GFP, PS1 or the dominant negative mutant PS1 D385A for 9–10 days. Then, HA-Nlg1 was expressed in isolated neurons by transfection and accumulation of neurexins to transfected dendrites was studied with the cyto-neurexin antibody. We reasoned that accumulation of neurexin-CTFs could be visualized as an increase in fluorescence staining detected with the cyto-neurexin antibody. Indeed, we observed increased cyto-neurexin staining at HA-Nlg1-mediated synapses in neurons expressing PS1 D385A, as compared to GFP or PS1-infected neurons (Fig 6A). Quantification analysis of multiple confocal images showed an increase in the relative dendritic area occupied by neurexin in neurons expressing PS1 D385A (Fig. 6C).

In the above experiments PS1 was overexpressed in both the pre- and postsynaptic neurons. To determine whether the accumulation of neurexins is due to a presynaptic effect of PS1 rather than an effect of PS1 in the postsynaptic cell, we adapted the mixed-culture assays described before to inhibit PS1 function selectively at axons contacting HA-Nlg1-expressing cells. For that, hippocampal neurons at 3 DIV were infected with lentiviral vectors expressing GFP, PS1 or PS1 D385A for 9–10 days and then co-cultured with HEK293 cells expressing HA-Nlg1. Thus, the expression of exogenous PS1 was restricted to axons at the time of synaptic differentiation, while the *postsynaptic* HEK293 cells expressing HA-Nlg1 were not affected. Again, we found that axonal expression of PS1 D385A increased the presynaptic recruitment of neurexins (Fig. 6B and 6D). Taken together, these studies indicate that PS regulate the processing and accumulation of neurexin-CTFs at presynaptic terminals of glutamatergic synapses.

## Discussion

Neurexins belong to a large family of plasma membrane proteins with synaptic function. In this study, we show that PS/γ-secretase mediates the cleavage of neurexins at synapses. Inhibition of PS/γ-secretase results in the accumulation of neurexin-CTFs *in vitro* and *in vivo*. Loss of PS function enhances neurexin accumulation at synaptic contacts mediated by HA-Nlg1, whereas expression of HA-Nlg1 triggers the recruitment of PS1 to glutamatergic terminals. Moreover, neurexin-CTFs mostly accumulate at presynaptic terminals of PS cDKO mice coinciding with initial synaptic and memory impairments and prior to the onset of neurodegeneration, suggesting that impaired neurexin processing caused by loss of PS function is an early event in the synaptic plasticity and memory deficits observed in PS cDKO mice.

Based on biochemical analyses, we demonstrate that neurexins are sequentially cleaved by metalloprotease- and PS/γ-secretase-dependent activities. Expression of epitope-tagged α- and β-neurexin isoforms in heterologous cells generates neurexin-CTFs, which abnormally accumulate in the presence of the PS/γ-secretase inhibitor DAPT. Immunoprecipitation of conditioned media of HEK293 cells transfected with HA-βNrx1 and treated with the metalloprotease inhibitor GM6001 revealed that neurexin-CTFs are generated after shedding of the extracellular domain. Interestingly, the ectodomain of APP is shed by α- and β-secretases at alternative sites within the extracellular region, yielding α-CTF and β-CTF fragments that are substrates of PS/γ-secretase [35]. In this line, we show that neurexin-CTFs appear as two distinct species of ∼12–15 kDa, suggesting the possibility that, similarly to APP, differential cleavage in the extracellular domain of neurexin generates CTFs of distinct sizes. Although the functional significance of neurexin ectodomain shedding by metalloprotease(s) requires further investigation and we can not rule out the involvement of other proteases, our results with the TAPI-1 inhibitor suggest a role of TACE/ADAM17 metalloprotease in the shedding of β-neurexins-1 and -2, as recently demonstrated for β-neurexin-3 [36].

Ectodomain shedding of type I proteins by metalloproteases is usually followed by regulated transmembrane cleavage by the γ-secretase complex [35]. It has been reported that inactivation of γ-secretase by genetic ablation or mutation of the aspartate residues within the active site of PS1 diminishes APP cleavage and Aβ generation, as well as the processing of other PS/γ-secretase substrates, such as Notch and N-cadherin [27], [39], [42]–[44]. Consistent with a role of PS/γ-secretase in the regulation of neurexin processing, we show that pharmacological or genetic inactivation of PS function by using DAPT or by expressing the dominant negative PS1 D385A mutant results in the accumulation of neurexin-CTFs in hippocampal neurons. Similarly, neurexin-CTFs levels are increased in brain lysates of both PS1−/− embryos and adult PS cDKO mice, confirming that neurexins are processed by PS/γ-secretase *in vivo*.

One of the key findings of our study is that neurexins are proteolytically processed by PS/γ-secretase at synapses, mostly at presynaptic terminals. It is worth noting that although neurexins were originally proposed as presynaptic proteins [45], immunohistochemical and electrophysiological studies have identified neurexin functions at both pre- and postsynaptic sites [11], [25], [46], [47]. In addition, neurexins accumulate intracellularly at the cell body of neurons [11]. Therefore, a relevant question regarding the regulation of neurexins by PS is to determine whether PS/γ-secretase mediates the processing of neurexins at synapses. By analyzing synaptosomal fractions from adult mice, we were able to detect low levels of neurexin-CTFs at the presynaptic fraction of control mice, which were robustly increased in PS cDKO mice. In addition, neurexin-CTFs were accumulated at lower levels in the postsynaptic fraction of PScDKO mice. The subcellular localization of neurexin-CTFs resembles that of APP CTFs, which accumulate mainly in presynaptic compartments in PS1cKO mice [38]. The fact that PS are deleted specifically in glutamatergic neurons in PS cDKO mice strongly suggests that the PS-dependent proteolysis of neurexins takes place at glutamatergic synapses, and lack of PS expression or function would result in accumulation of neurexin-CTFs at glutamatergic synapses. Accordingly, we found that loss of PS1 function by expression of the PS1 D385A mutant increases neurexin signal at glutamatergic terminals induced by HA-Nlg1. This effect is likely mediated by presynaptic PS, as suggested by our experiments where the function of PS1 was inhibited in axons contacting HA-Nlg1 expressing HEK293 cells. Importantly, we found that co-culture of hippocampal neurons with HEK293 cells expressing HA-Nlg1 recruits PS1 to glutamatergic terminals, thus concentrating PS1 within the same synaptic compartment as β-neurexins. Since β-neurexins and HA-Nlg1 participate in a trans-synaptic mechanism that mediates glutamatergic differentiation [12], [16]–[19], [48], our results suggest that PS-mediated neurexin processing could affect glutamatergic synapse assembly and/or function.

We have recently shown that PS expression in postnatal mouse brain is required for maintaining normal synaptic function and memory [5]. Accordingly, conditional inactivation of both PS genes in forebrain glutamatergic neurons results in synaptic and memory deficits in PS cDKO mice at 2 months of age, which are followed by progressive neurodegeneration at 4–6 months [5], [49]. The early synaptic deficits in PS cDKO mice are associated with impaired paired-pulse facilitation and NMDA receptor-mediated responses, and long-term potentiation (LTP) deficits [5]. These deficits are likely caused by a PS-dependent presynaptic mechanism. Thus, PS expressed presynaptically, but not postsynaptically, are required for neurotransmitter release and induction of LTP in Schaffer-collateral pathway synapses [6]. These observations indicate that PS acting by a presynaptic mechanism regulate glutamatergic neurotransmitter release and LTP induction and suggest that impaired proteolysis of PS substrates may contribute to the synaptic defects caused by loss of PS function. It is noteworthy that the cytoplasmic tail of neurexins associates with a molecular complex formed by CASK and components of the neurotransmitter release machinery [50], and that presynaptic β-neurexins are required for maintaining the release probability at neuroligin-1 synapses in CA3 hippocampal neurons [51]. Our data showing that neurexin processing is mostly impaired in presynaptic terminals of PS cDKO mice suggest that neurexins are potential candidates to mediate the synaptic defects associated with loss of PS function. Additionally, the processing of neurexins might regulate the function of the postsynapses by a trans-synaptic mechanism mediated by neuroligins. The accumulation of neurexin-CTF at synaptosomes of PScDKO mice suggests the possibility that the proteolytic shedding of neurexin ectodomain can take place at glutamatergic synapses and, thereby, it may affect the binding with neuroligins located at the postsynaptic terminal.

More than 180 autosomal dominant inherited mutations in the PS genes are responsible for the majority of early-onset familial FAD cases (http://www.molgen.ua.ac.be/ADMutations). Mutations in PS alter the γ-secretase-dependent cleavage of APP, resulting in altered levels of amyloidogenic Aβ peptides. PS pathogenic mutations have distinct effects on the γ - and/or ε-secretase-dependent cleavages of different substrates including Notch, APP and N-Cadherin [39], [44], [52]. These data have lead to the hypothesis that FAD-linked PS mutations may act through a partial loss of function mechanism [7]. Notably, here we show that some FAD-linked *PS1* mutations are not able to rescue the normal processing of β-neurexin-1 when expressed in a PS null background. Specifically, FAD-linked mutations with the lowest levels of endoproteolysis, C410Y and ΔE9, fail to rescue the normal processing of β-neurexin-1. Some FAD-linked mutations, including C410Y and ΔE9, have been previously shown to negatively affect PS1 endoproteolysis or they alter the precise presenilinase cleavage of PS1 [53], although the mutant PS1 proteins are incorporated into the γ-secretase complex [40], [44], [54], [55]. In agreement with this result, we found that the maturation of nicastrin is increased in PS−/− cells expressing H163R, C410Y and ΔE9 mutants, although at lower levels than PS1 WT and M146L proteins. We therefore suggest that there is a differential effect of FAD-linked PS1 mutations on γ-secretase-dependent neurexin processing, as it is the case for other substrates. The relationship between PS endoproteolysis and PS/γ-secretase activity is still controversial. For instance, there is evidence that cleavage of PS and formation of heterodimeric PS NTFs and CTFs complexes is required for complete stabilization and function of PS/γ-secretase [24], [27], [56]. By contrast, other reports indicate that the amyloidogenic function of γ-secretase seems to be independent of PS endoproteolysis [57]. While this manuscript was in preparation, Bot et al. reported the shedding of β-neurexin-3 by α- and γ-secretase activities in heterologous cells [36]. Our results showing the processing of different α- and β-neurexin isoforms are consistent with a more general mechanism of neurexin shedding by PS, and importantly, demonstrate that such processing occurs at synapses during synaptic assembly. Finally, it is worth mentioning that the previous identification of mutations in the *NRXN1* gene affecting α and β isoforms associated with autism, mental retardation and schizophrenia strongly suggests a role for synaptic defects mediated by neurexins in mental diseases [23]. The data reported here showing the processing of β-neurexin-1 and the effect of FAD-linked *PS1* mutations could point at a dysfunction of neurexins in a broader range of brain disorders.

## Supporting Information

Figure S1 **Colocalization of PS1 with synaptic markers at HA-Nlg1-induced synapses.** Confocal images of cultured hippocampal neurons transfected with HA-Nlg1 and co-stained with HA antibody (green in the colocalization), PS1 N-terminal antibody (red in the colocalization) and the presynaptic marker synaptobrevin (VAMP2 antibody, blue in the colocalization) (upper panels) or the postsynaptic marker PSD95 (blue in the colocalization) (lower panels). Arrows indicate colocalization of PS1 with synaptic markers at HA-Nlg1-mediated synapses. Scale bar: 5 µm.
(TIF)

## References

[1] Koo EH, Kopan R (2004). Potential role of presenilin-regulated signaling pathways in sporadic neurodegeneration.. Nat Med.

[2] Selkoe DJ, Wolfe MS (2007). Presenilin: running with scissors in the membrane.. Cell.

[3] Hardy J, Selkoe DJ (2002). The amyloid hypothesis of Alzheimer's disease: progress and problems on the road to therapeutics.. Science.

[4] De Strooper B (2007). Loss-of-function presenilin mutations in Alzheimer disease. Talking Point on the role of presenilin mutations in Alzheimer disease.. EMBO Rep.

[5] Saura CA, Choi SY, Beglopoulos V, Malkani S, Zhang D (2004). Loss of presenilin function causes impairments of memory and synaptic plasticity followed by age-dependent neurodegeneration.. Neuron.

[6] Zhang C, Wu B, Beglopoulos V, Wines-Samuelson M, Zhang D (2009). Presenilins are essential for regulating neurotransmitter release.. Nature.

[7] Shen J, Kelleher RJ (2007). The presenilin hypothesis of Alzheimer's disease: evidence for a loss-of-function pathogenic mechanism.. Proc Natl Acad Sci U S A.

[8] Ullrich B, Ushkaryov YA, Sudhof TC (1995). Cartography of neurexins: more than 1000 isoforms generated by alternative splicing and expressed in distinct subsets of neurons.. Neuron.

[9] de Wit J, Sylwestrak E, O'Sullivan ML, Otto S, Tiglio K (2009). LRRTM2 interacts with Neurexin1 and regulates excitatory synapse formation.. Neuron.

[10] Ko J, Fuccillo MV, Malenka RC, Sudhof TC (2009). LRRTM2 functions as a neurexin ligand in promoting excitatory synapse formation.. Neuron.

[11] Dean C, Scholl FG, Choih J, DeMaria S, Berger J (2003). Neurexin mediates the assembly of presynaptic terminals.. Nat Neurosci.

[12] Boucard AA, Chubykin AA, Comoletti D, Taylor P, Sudhof TC (2005). A splice code for trans-synaptic cell adhesion mediated by binding of neuroligin 1 to alpha- and beta-neurexins.. Neuron.

[13] Scheiffele P, Fan J, Choih J, Fetter R, Serafini T (2000). Neuroligin expressed in nonneuronal cells triggers presynaptic development in contacting axons.. Cell.

[14] Siddiqui TJ, Pancaroglu R, Kang Y, Rooyakkers A, Craig AM (2010). LRRTMs and neuroligins bind neurexins with a differential code to cooperate in glutamate synapse development.. J Neurosci.

[15] Uemura T, Lee SJ, Yasumura M, Takeuchi T, Yoshida T (2010). Trans-synaptic interaction of GluRdelta2 and Neurexin through Cbln1 mediates synapse formation in the cerebellum.. Cell.

[16] Chih B, Gollan L, Scheiffele P (2006). Alternative splicing controls selective trans-synaptic interactions of the neuroligin-neurexin complex.. Neuron.

[17] Song JY, Ichtchenko K, Sudhof TC, Brose N (1999). Neuroligin 1 is a postsynaptic cell-adhesion molecule of excitatory synapses.. Proc Natl Acad Sci U S A.

[18] Graf ER, Zhang X, Jin SX, Linhoff MW, Craig AM (2004). Neurexins induce differentiation of GABA and glutamate postsynaptic specializations via neuroligins.. Cell.

[19] Varoqueaux F, Jamain S, Brose N (2004). Neuroligin 2 is exclusively localized to inhibitory synapses.. Eur J Cell Biol.

[20] Marshall CR, Noor A, Vincent JB, Lionel AC, Feuk L (2008). Structural variation of chromosomes in autism spectrum disorder.. Am J Hum Genet.

[21] Szatmari P, Paterson AD, Zwaigenbaum L, Roberts W, Brian J (2007). Mapping autism risk loci using genetic linkage and chromosomal rearrangements.. Nat Genet.

[22] Kim HG, Kishikawa S, Higgins AW, Seong IS, Donovan DJ (2008). Disruption of neurexin 1 associated with autism spectrum disorder.. Am J Hum Genet.

[23] Sudhof TC (2008). Neuroligins and neurexins link synaptic function to cognitive disease.. Nature.

[24] Saura CA, Tomita T, Davenport F, Harris CL, Iwatsubo T (1999). Evidence that intramolecular associations between presenilin domains are obligatory for endoproteolytic processing.. J Biol Chem.

[25] Taniguchi H, Gollan L, Scholl FG, Mahadomrongkul V, Dobler E (2007). Silencing of neuroligin function by postsynaptic neurexins.. J Neurosci.

[26] Saura CA, Tomita T, Soriano S, Takahashi M, Leem JY (2000). The nonconserved hydrophilic loop domain of presenilin (PS) is not required for PS endoproteolysis or enhanced abeta 42 production mediated by familial early onset Alzheimer's disease-linked PS variants.. J Biol Chem.

[27] Wolfe MS, Xia W, Ostaszewski BL, Diehl TS, Kimberly WT (1999). Two transmembrane aspartates in presenilin-1 required for presenilin endoproteolysis and gamma-secretase activity.. Nature.

[28] Gascon S, Paez-Gomez JA, Diaz-Guerra M, Scheiffele P, Scholl FG (2008). Dual-promoter lentiviral vectors for constitutive and regulated gene expression in neurons.. J Neurosci Methods.

[29] Lois C, Hong EJ, Pease S, Brown EJ, Baltimore D (2002). Germline transmission and tissue-specific expression of transgenes delivered by lentiviral vectors.. Science.

[30] Herreman A, Serneels L, Annaert W, Collen D, Schoonjans L (2000). Total inactivation of gamma-secretase activity in presenilin-deficient embryonic stem cells.. Nat Cell Biol.

[31] Shen J, Bronson RT, Chen DF, Xia W, Selkoe DJ (1997). Skeletal and CNS defects in Presenilin-1-deficient mice.. Cell.

[32] Peng J, Kim MJ, Cheng D, Duong DM, Gygi SP (2004). Semiquantitative proteomic analysis of rat forebrain postsynaptic density fractions by mass spectrometry.. J Biol Chem.

[33] Cho KO, Hunt CA, Kennedy MB (1992). The rat brain postsynaptic density fraction contains a homolog of the Drosophila discs-large tumor suppressor protein.. Neuron.

[34] Winkler E, Hobson S, Fukumori A, Dumpelfeld B, Luebbers T (2009). Purification, pharmacological modulation, and biochemical characterization of interactors of endogenous human gamma-secretase.. Biochemistry.

[35] Wolfe MS, Kopan R (2004). Intramembrane proteolysis: theme and variations.. Science.

[36] Bot N, Schweizer C, Ben Halima S, Fraering PC (2011). Processing of the synaptic cell-adhesion molecule neurexin-3beta by Alzheimer's disease alpha- and gamma-secretases.. J Biol Chem.

[37] Yu H, Saura CA, Choi SY, Sun LD, Yang X (2001). APP processing and synaptic plasticity in presenilin-1 conditional knockout mice.. Neuron.

[38] Saura CA, Chen G, Malkani S, Choi SY, Takahashi RH (2005). Conditional inactivation of presenilin-1 prevents amyloid accumulation and temporarily rescues contextual and spatial working memory impairments in amyloid precursor protein transgenic mice.. J Neurosci.

[39] Marambaud P, Wen PH, Dutt A, Shioi J, Takashima A (2003). A CBP binding transcriptional repressor produced by the PS1/epsilon-cleavage of N-cadherin is inhibited by PS1 FAD mutations.. Cell.

[40] Steiner H, Romig H, Grim MG, Philipp U, Pesold B (1999). The biological and pathological function of the presenilin-1 Deltaexon 9 mutation is independent of its defect to undergo proteolytic processing.. J Biol Chem.

[41] Biederer T, Scheiffele P (2007). Mixed-culture assays for analyzing neuronal synapse formation.. Nat Protoc.

[42] De Strooper B, Saftig P, Craessaerts K, Vanderstichele H, Guhde G (1998). Deficiency of presenilin-1 inhibits the normal cleavage of amyloid precursor protein.. Nature.

[43] De Strooper B, Annaert W, Cupers P, Saftig P, Craessaerts K (1999). A presenilin-1-dependent gamma-secretase-like protease mediates release of Notch intracellular domain.. Nature.

[44] Bentahir M, Nyabi O, Verhamme J, Tolia A, Horre K (2006). Presenilin clinical mutations can affect gamma-secretase activity by different mechanisms.. J Neurochem.

[45] Ushkaryov YA, Petrenko AG, Geppert M, Sudhof TC (1992). Neurexins: synaptic cell surface proteins related to the alpha-latrotoxin receptor and laminin.. Science.

[46] Kattenstroth G, Tantalaki E, Sudhof TC, Gottmann K, Missler M (2004). Postsynaptic N-methyl-D-aspartate receptor function requires alpha-neurexins.. Proc Natl Acad Sci U S A.

[47] Missler M, Zhang W, Rohlmann A, Kattenstroth G, Hammer RE (2003). Alpha-neurexins couple Ca2+ channels to synaptic vesicle exocytosis.. Nature.

[48] Lee H, Dean C, Isacoff E (2010). Alternative splicing of neuroligin regulates the rate of presynaptic differentiation.. J Neurosci.

[49] Wines-Samuelson M, Schulte EC, Smith MJ, Aoki C, Liu X (2010). Characterization of age-dependent and progressive cortical neuronal degeneration in presenilin conditional mutant mice.. PLoS One.

[50] Butz S, Okamoto M, Sudhof TC (1998). A tripartite protein complex with the potential to couple synaptic vesicle exocytosis to cell adhesion in brain.. Cell.

[51] Futai K, Kim MJ, Hashikawa T, Scheiffele P, Sheng M (2007). Retrograde modulation of presynaptic release probability through signaling mediated by PSD-95-neuroligin.. Nat Neurosci.

[52] Song W, Nadeau P, Yuan M, Yang X, Shen J (1999). Proteolytic release and nuclear translocation of Notch-1 are induced by presenilin-1 and impaired by pathogenic presenilin-1 mutations.. Proc Natl Acad Sci U S A.

[53] Fukumori A, Fluhrer R, Steiner H, Haass C (2010). Three-amino acid spacing of presenilin endoproteolysis suggests a general stepwise cleavage of gamma-secretase-mediated intramembrane proteolysis.. J Neurosci.

[54] Murayama O, Murayama M, Honda T, Sun X, Nihonmatsu N (1999). Twenty-nine missense mutations linked with familial Alzheimer's disease alter the processing of presenilin 1.. Prog Neuropsychopharmacol Biol Psychiatry.

[55] Thinakaran G, Borchelt DR, Lee MK, Slunt HH, Spitzer L (1996). Endoproteolysis of presenilin 1 and accumulation of processed derivatives in vivo.. Neuron.

[56] Takasugi N, Tomita T, Hayashi I, Tsuruoka M, Niimura M (2003). The role of presenilin cofactors in the gamma-secretase complex.. Nature.

[57] Steiner H, Romig H, Pesold B, Philipp U, Baader M (1999). Amyloidogenic function of the Alzheimer's disease-associated presenilin 1 in the absence of endoproteolysis.. Biochemistry.

