# Supplementary figures and images for: Presenilin/γ-Secretase Regulates Neurexin Processing at Synapses
Source: PLoS One. 2011 Apr 29;6(4):e19430. doi: 10.1371/journal.pone.0019430 (PMC3084856; doi:10.1371/journal.pone.0019430)

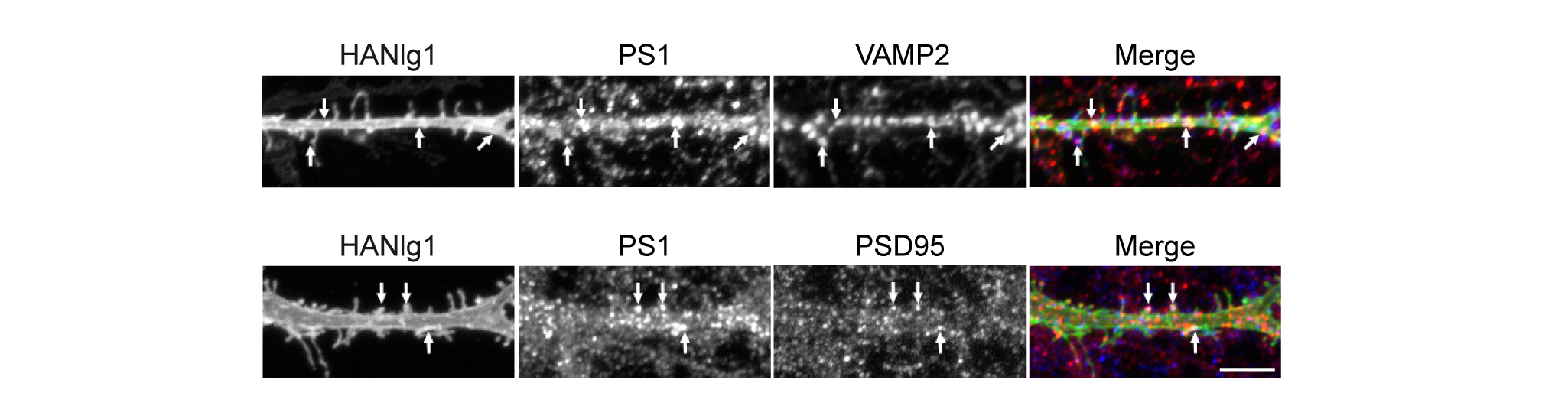

Supplement: Figure S1 — Colocalization of PS1 with synaptic markers at HA-Nlg1-induced synapses. Confocal images of cultured hippocampal neurons transfected with HA-Nlg1 and co-stained with HA antibody (green in the colocalization), PS1 N-terminal antibody (red in the colocalization) and the presynaptic marker synaptobrevin (VAMP2 antibody, blue in the colocalization) (upper panels) or the postsynaptic marker PSD95 (blue in the colocalization) (lower panels). Arrows indicate colocalization of PS1 with synaptic markers at HA-Nlg1-mediated synapses. Scale bar: 5 µm. (TIF) [file pone.0019430.s001.tif]
